# Wolf risk fails to inspire fear in two mesocarnivores suggesting facilitation prevails

**DOI:** 10.1038/s41598-022-20725-3

**Published:** 2022-10-01

**Authors:** Tom A. Diserens, Marcin Churski, Jakub W. Bubnicki, Andrzej Zalewski, Marcin Brzeziński, Dries P. J. Kuijper

**Affiliations:** 1 Mammal Research Institute, Polish Academy of Sciences, Ul. Stoczek 1, 17-230 Białowieża, Poland; 2 Faculty of Biology, University of Warsaw, Ul. Miecznikowa 1, 02‑097 Warsaw, Poland

**Keywords:** Ecology, Behavioural ecology

## Abstract

Large carnivores not only supress mesocarnivores via killing and instilling fear, but also facilitate them through carrion provisioning. Hence, mesocarnivores frequently face a trade-off between risk avoidance and food acquisition. Here we used the raccoon dog and red fox in Białowieża Forest, Poland as models for investigating how large carnivores shape mesocarnivore foraging behaviour in an area with widespread large carnivore carrion provisioning. Using a giving up density experiment we quantified mesocarnivore foraging responses to wolf body odour across a landscape-scale gradient in wolf encounter rates. At locations with higher wolf encounter rates, raccoon dogs depleted feeding trays more than at plots with lower wolf encounter rates. Simulating wolf presence by adding wolf body odour caused raccoon dogs to deplete feeding trays more at locations with low wolf encounter rates, but less at locations with high wolf encounter rates. Fox foraging costs did not vary with the application of wolf body odour or wolf encounter rates. The frequency that the mesocarnivores visited experimental foraging patches was unaffected by wolf body odour or landscape level encounter rates. These results provide further evidence that large carnivore suppression can play a subordinate role to facilitation in determining mesocarnivore behaviour. The varying raccoon dog response to wolf odour across the landscape-scale gradient in wolf encounter rates shows how mesocarnivore-large carnivore interactions can be context-dependent. We suggest that rather than testing the effects of single risk cues on prey behaviour, future studies should focus on understanding how context modifies the ecological impacts of large carnivores.

## Introduction

Large, competitively dominant carnivores can supress the abundances of sympatric mesocarnivores^1–3^. To reduce their chances of being killed, the latter can change their behaviour in response to risk^4–6^. However, besides posing a threat, large carnivores can also facilitate mesocarnivores through carrion provisioning^3,7^. As scavenging tends to be risky due to the possibility of direct encounters with large carnivores at kill sites, mesocarnivores frequently face a trade-off between food acquisition and risk avoidance^4,8^. To make optimal foraging decisions and avoid encounters with their predators, mesocarnivores must assess how predation risk varies in space and time. Despite the importance of large carnivores for biodiversity and ecosystem functioning^9^, we still know little about how they affect mesocarnivores and particularly their behaviour in Europe^10^.

Space use by large carnivores creates areas in the landscape that mesocarnivores perceive as high or low risk (the ‘landscape of fear’, e.g.^11^), which can cause behavioural changes in the latter^4,12^ that can cascade down food chains^9,13^. In response to variation in perceived risk, mesocarnivores can reduce their probability of being predated by changing a variety of behaviours such as habitat use, temporal niches, vigilance, and spatial patterns^12^. They have been found to, inter alia, reduce or delay their foraging^14–16^ and increase their vigilance in response to predator cues^15,17,18^, and change their space use to avoid areas where large carnivores are perceived to be abundant^5,6,19,20^. However, the mesocarnivore behavioural response to large carnivore risk is species and context dependent^10,21^, and species can respond to the same risk cue differently, even oppositely, depending on how, when and where it was deposited^22^. Several contextual factors can modify prey responses to risk, including, amongst others, (i) carnivore guild composition and (ii) carnivore densities (iii) habitat structure and (iv) food availability^10,21^. Recent studies have found food availability to be especially important in determining mesocarnivore responses to risk^23,24^. Carrion provisioning can cause large carnivore presence (or their cues) to indicate both risk and reward^24^, and therefore the peaks in the landscapes of fear and food to overlap^8,12^. Consequently, mesocarnivores can show counterintuitive behavioural responses to areas with high levels of risk^23,24^, whereby they are attracted to large carnivore kill sites to scavenge^7^ despite kill sites being hotspots of large carnivore activity^25–27^. Mesocarnivores persist in visiting kill sites, presumably because the benefits from scavenging outweigh the costs of the predation risk. It is noteworthy that the literature refers to the benefits that mesocarnivores derive from kleptoparasitism as facilitation^3,23,24^, implying no harm is done to the provider of the carcass; yet in practice this is unlikely to be the case, as large carnivores usually lose resources in this interaction. Still, we know little about how facilitation in the form of carrion provisioning modifies the effect of predation risk on mesocarnivore behaviour, or about how the context determines the balance between these opposing ecological processes.

In this study, to explore how large carnivores suppress mesocarnivore behaviour in a landscape with widespread large carnivore carrion provisioning^7,25^, we investigated how wolf *Canis lupus* risk affects raccoon dog *Nyctereutes procyonoides* and red fox *Vulpes vulpes* foraging decisions in Białowieża Forest (BF), Poland. Wolves kill raccoon dogs and foxes^28,29^ but also facilitate them through carrion provisioning^7,25^. Thus, we predicted wolves would both inspire fear in and attract these mesocarnivores in BF, making this system a model for studying how large carnivore suppressive and facilitative effects shape mesocarnivore behaviour. We used giving up densities (GUDs) to quantify the trade-off between risk-avoidance and foraging^30^. GUDs are a method for quantifying an animal’s perceived costs of foraging at experimental feeding patches^30,31^. As a forager depletes a patch, the density of food decreases, as does the harvest rate^30^. The amount of food remaining in a patch when an animal ceases foraging, the GUD value, represents the food density at which foraging is no longer worthwhile^30,31^. We quantified mesocarnivore responses at experimental foraging patches exposed to wolf odour across a natural landscape-scale gradient in wolf encounter rates, proxies for fine-scale and landscape level risk, respectively. If suppression effects are present, we expected mesocarnivores would (1) perceive higher costs of foraging at and (2) show lower use of study plots with wolf odour (relative to plots without odour) and/or plots with higher wolf encounter rates (suppression hypotheses). However, widespread carrion provisioning in BF may cause mesocarnivores to associate large carnivore cues with reward rather than risk and so suppression may not be present. Thus, we alternatively expected mesocarnivores would (3) not perceive higher costs of foraging at and (4) show the same or higher use of plots with wolf odour (relative to plots without odour) and/or plots with higher wolf encounter rates (facilitation hypotheses). We also investigated the interaction between wolf encounter rates and body odour to test whether the effect of simulated wolf presence varies with the landscape level risk context. If the response to wolf body odour is context dependent, our hypotheses would hold true in only part of the landscape-scale gradient in wolf encounter rates. We discuss how the context and presence of facilitative effects can modify how large carnivore risk affects mesocarnivore behaviour.

## Materials and methods

### Study area

**Figure 1:**
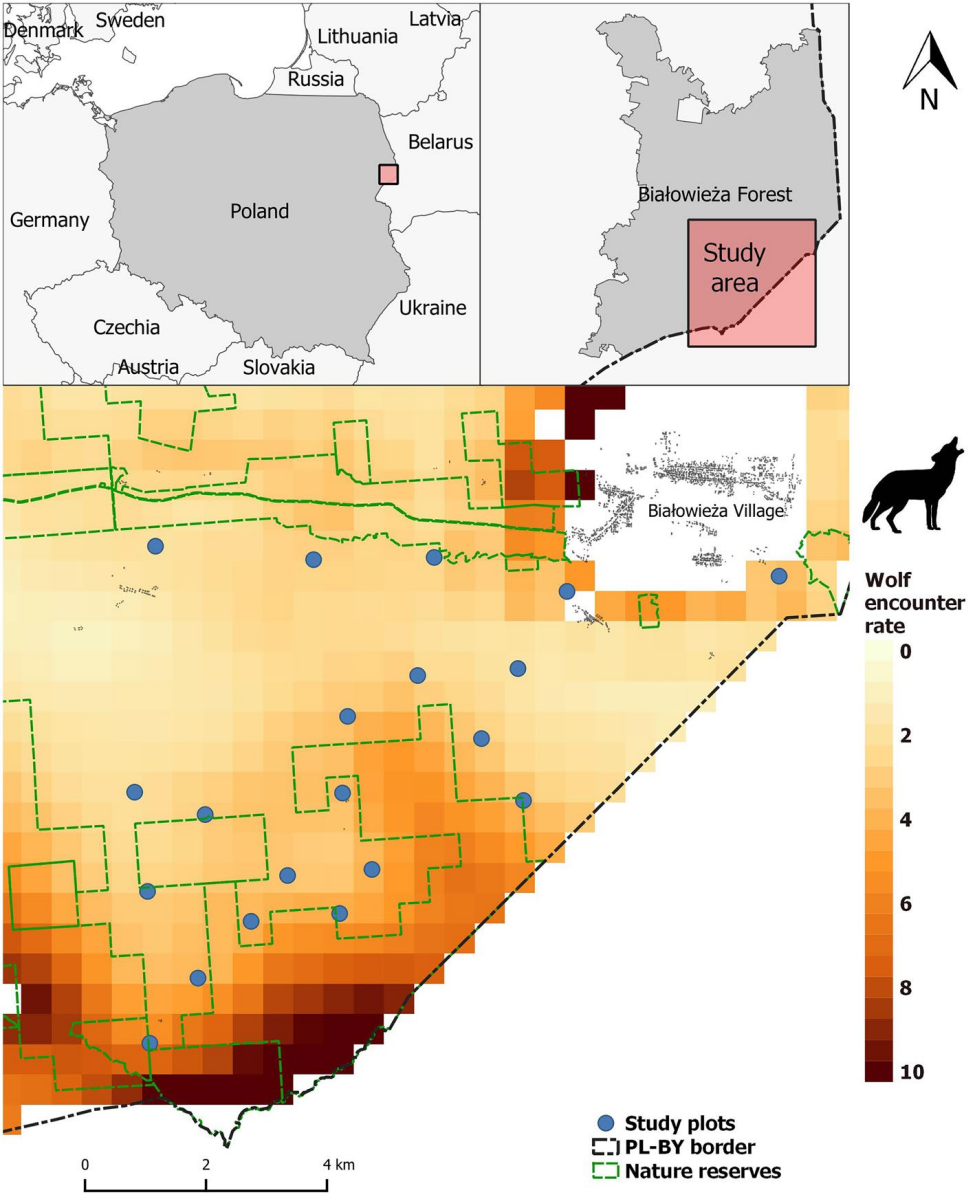
The locations of the 20 plots studied in the southeast of the Polish BF overlaid with the wolf encounter rate gradient, from light orange (lowest risk) to dark red (highest risk). Wolf encounter rates refer to the patterns of space use of wolves in 500 × 500 m grid cells (as determined in Bubnicki et al.^34^). We generated the image in QGIS v 3.16.16 (www.qgis.org).

BF, one of the best-preserved forests in the European lowlands, spans the Polish-Belarusian border (52°30′–53°N, 23°30′–24°15′E) and covers 1450 km^2^, with 600 km^2^ in Poland and 850 km^2^ in Belarus. The area is relatively flat (134–197 m a.s.l.), and its climate is transitional between Atlantic and continental types, with a mean annual temperature of 7 °C and precipitation of 641 mm. Our study took place on the Polish side, whose forest habitats comprise oak–lime–hornbeam forest (56.5%), wet ash–alder forest (19.2%), and coniferous and mixed forest (17.9%), with open habitats covering the rest of the area (glades with meadows, riverside open sedge and reed marshes − 6.4%) (calculated from^32^). The best-preserved stands are protected within Białowieża National Park, covering 18% (105 km^2^) of the Polish side, where hunting and forestry is banned. The rest is managed by the Polish State Forest Holding and subject to small-scale clear cuts and hunting, but where also exists a network of nature reserves that protects well-preserved old-growth stands (22%, ca. 130 km^2^). A temporary ban on forestry meant that no logging occurred in BF over the field work period^33^. The study plots were all located in the southeastern part of the Polish BF, outside the national park and nature reserves (Fig. 1), where permissions are not required for conducting this type of research. This area is freely accessible to hikers and cyclists, and only accessible to cars with a permit. To keep the habitat type in the vicinity of the study plots similar, we restricted the study plots to the more fertile, deciduous habitat types.

### Carnivore community and known interactions

BF hosts a rich carnivore community (by European standards) with 12 carnivore species^24^. Among them, the study focussed on two terrestrial mesocarnivores, the red fox, and invasive raccoon dog, which has occurred in the forest since the 1950s. In BF, they occur at average densities up to 5 raccoon dogs, and 3.5 foxes per 10 km^2^ in their most preferred habitat^25^, and both species are subject to hunting harvest. We had initially aimed to also study the European badger *Meles meles*, and pine marten *Martes martes,* but these visited our plots infrequently and so were excluded from the analysis; they likely visited our plots less frequently due to their occurring at lower densities (in the case of the badger, which occurs at up to 1.57 individuals per 10 km^2^^35^), the availability of alternative food (badgers specialise on earthworms, pine marten on rodents) and have different ecologies (neither specialise in scavenging, in contrast to the raccoon dog and fox)^25^. In BF these mesocarnivores coexist with two large carnivores, the wolf and lynx (*Lynx lynx*), which have been strictly protected and not hunted on the Polish side of BF since the 1990s. In this system, predation is responsible for 27% of raccoon dog mortality, with domestic dogs *Canis lupus familiaris* responsible for 9%, wolves 7%, unidentified predators 7%, and other predators the remaining 4%^28^. Data on the causes of fox mortality in BF are unavailable, but wolves are generally known to kill foxes^29^, and foxes occasionally appear in the wolf diet^25^. The studied mesocarnivores are generalist omnivores, and in BF ungulate carcasses are important components in both their diets, comprising 29% of biomass of the raccoon dog’s diet in spring–summer, and 56% in autumn–winter, and ca. 25% of the fox’s in both seasons^25^. They regularly track wolves to find carrion for sustenance^7,25^, with raccoon dogs kleptoparasitizing 40% of wolf kills, and foxes 87%^7^. The home ranges of wolves and lynx cover the entire forest complex^36,37^, but they show clear gradients in space-use; hence the probability of encountering them varies across the landscape^34^. Space use by wolves and lynx overlaps to a large degree^34^ and they do not spatially avoid each other^37^. Three to four wolf packs (each with 5–12 individuals) occur on the Polish side of BF^34,36^. A recent study found that the lynx occurs at much lower densities than the wolf^34^. For this reason, in the present study we chose to only study the effects of wolf risk on mesocarnivore behaviour. A brown bear *Ursus arctos* was documented in the forest over the field study period^34^. However, there have been no sightings of females with cubs, suggesting it is too early to declare the species as having recolonized BF.

### Sourcing and storing the wolf body odour

As an experimental cue indicating risk we used fresh wolf body odour, intending to simulate the possible close proximity of a wolf. Studies have demonstrated that predator body odour can elicit a clear fear response in prey species^14,38,39^. While other studies have shown body odour elicits stronger fear responses in prey species than faecal and excretory odours^40,41^. Body odour has been suggested to have more direct link to immediate predator presence, and pose a more imminent threat to prey^40,41^. We refreshed the odour at study plots each study day, as studies have found prey antipredator responses can diminish as predator cues age^22,38^. To collect the wolf body odour, we used brown 100% cotton towels, which we washed in a washing machine without detergent and dried on a line outside prior to use. We designated half the towels as control towels (no odour), and half as wolf odour towels (to be soaked in wolf body-odour). The control towels were left on the washing line outside, while the treatment towels were deposited in the sleeping area of a captive female adult wolf for 21 days. As in Leo et al.^14^, we chose this period to ensure the towels absorbed the smell of the wolf in a way as to represent its repeated visits to a rendezvous site. The wolf was unsterilized, had lived apart from other captive wolves for at least three years, and was isolated from wild conspecifics by a fence. The collected towels were cut into 10 × 10 cm pieces, which were packed into plastic bags and placed in a freezer set at – 80 °C. The control and treatment towels were kept apart at all times and only handled with clean plastic lab gloves to avoid contamination. To ensure freshness of the scent, the body-odour towels were outside of the wolf pen and freezer for less than 5 h before being taken into the field, and were used within three months of freezing.

### Quantifying landscape-scale gradient in wolf encounter rates

The gradient in landscape level wolf encounter rates in the study area was obtained from a recent study^42^ (for a visualisation of the risk gradient see Fig. 1). Briefly, the study entailed deploying 73 camera traps along forest tracks for one month in September–October 2015. Seasonal changes in territory use do not change the overall pattern of wolf space use in BF. Throughout the year the wolf packs move widely throughout their territories, but return to the cores of their territories regularly, spending most of their time there (see^36,43,44^). Thus the chosen monitoring period gave a representative, ‘averaged’ picture of the relative annual use of the BF landscape by wolves. The sampling strategy was designed to capture and model the fine-scale, continuous variation in wolf landscape level space use. A hierarchical multi-scale spatial model was developed to show the intensity of wolf space use in 500 × 500 m grid cells across the whole forest. In the present study, we assumed that higher wolf encounter rates translated into higher perceived predation risk for mesocarnivores. Although the spatial data for wolves comes from 2015, and our study was carried out in 2019, we do not believe this temporal mismatch to be an issue. Since 1998, 3–4 wolf packs have inhabited BF^36^ and their territory and core activity areas have remained relatively stable over the past two decades (see^34^). The southeastern part of the Polish BF encompasses a gradient of wolf activity, with the nature reserves adjacent to the Polish-Belarusian border an area with high wolf activity where one wolf pack usually locates its den^34,45^. Wolf activity gradually decreases moving northwards, away from the nature reserves^34,45^. As we did not work in nature reserves, we may have missed putting study plots in the immediate vicinity of a wolf den, which is where the indirect effects of wolves have been observed to be strongest^46^. However, the aim of the paper was to investigate the mesocarnivore response along a gradient of wolf risk at a landscape scale.

### Giving up density field methods

We carried out a brief pilot study to determine the best set up for the GUD experiment. Its aim was to reveal the following: (i) a bait that would draw the mesocarnivores to the study plots, (ii) a cost-effective universal type of food that all the mesocarnivore species would readily eat, (iii) an optimal neutral substrate that would be both diggable for the mesocarnivores and easy enough to sift through by a field worker when counting the remaining food pieces, and (iv) the optimal ratio of food to neutral substrate^31^, (v) locations where the target species are definitely present in the vicinity.

**Figure 2:**
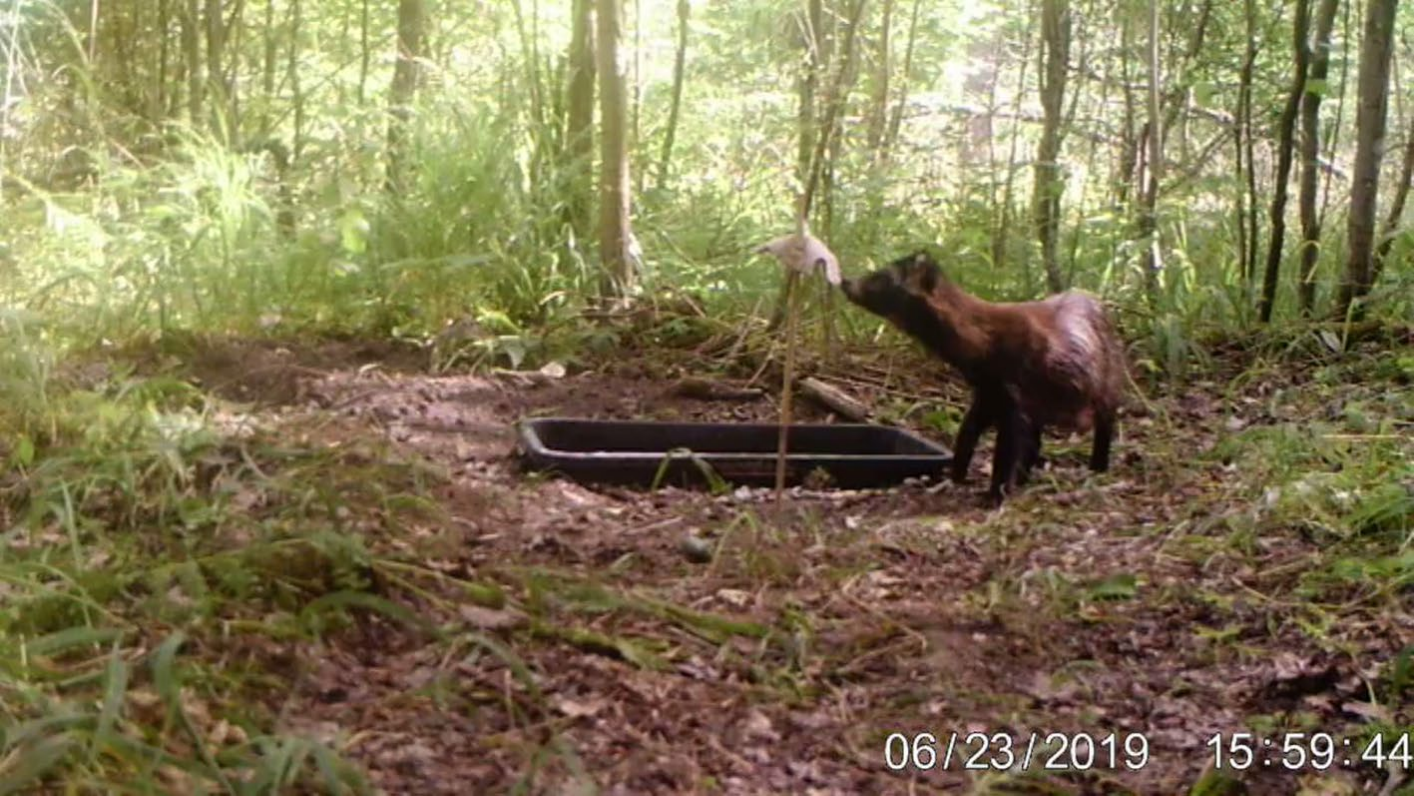
A still frame from a camera trap video showing our experimental set up. The picture shows a raccoon dog interacting with a treatment towel on the end of the bamboo stick with a feeding tray half buried in the ground adjacent.

We chose 20 locations (plot hereinafter) a minimum of ca. 1 km apart (Fig. 1). The plots were inside the tree stands 10–20 m away from forest roads. Each plot comprised a 40 L rectangular black plastic tray (70 × 40 cm) placed into a shallow hole in the ground, with a distance between the tray rim and the ground of 5–10 cm, see Fig. 2. Trays were filled to ca. 5 cm from the top with gardening gravel (grain size 8-16 mm). We drilled several holes into the trays so rain water could drain out. On study days the gravel contained, evenly mixed into it, 20 dry dog food pellets (Chappi brand) comprising a mix of cereals, vegetables, fats and meat. We do not believe this food to substrate ratio to have only permitted the observation of strong responses, as foxes and raccoon dogs ate anywhere from 0 to 19 pieces regularly, indicating the animals’ clear ability to dig to the bottom of the gravel. At each station we deployed one camera trap (LTL Acorn SGN-5310 M) on a tree 5–10 m away from the tray to identify animals visiting the trays and record their behaviour. The cameras were set to record 60 s videos at 0 s intervals upon being triggered by motion. Within 0.5 m of the trays, each station was equipped with a bamboo cane upon which the odour towels were attached with bamboo clothes pegs. In the first three sessions the canes were 50 cm long, but due to animals repeatedly interacting with them, we used 1 m long ones in the final three sessions.

We carried out the study in 2019 during three periods: Study Period 1—early summer (June–July), Study Period 2—late summer (August–September), and Study Period 3—autumn (October). The field work was timed to span the vegetative season (May–October), as this is when the mesocarnivores are most active, and when food is abundant in the forest and thus when risk effects should be strongest^8^. We carried out two sessions in each period, with ten of the twenty plots studied per session. Plots were randomly assigned to one of the two sessions in each period, so that each plot was studied once per period. Sessions varied in length from 5 to 11 days for logistical reasons. During each session five plots were subject to the wolf odour treatment, and five were controls. Plots received alternative treatments from study period to study period; if a plot received wolf body odour treatment in period 1, it then received the control in period 2, and so on. The study periods had a minimum of 10 days gap between them, and we assumed that this was sufficient time to prevent the treatment applied during one period affecting the results of the subsequent study period. Prior to the experiment, we carried out a two week habituation phase for the animals to get used to the presence of the trays and food. During this phase plots were visited daily and the food replenished if any was eaten; no wolf body odour was left at this point. The feeding trays were left in the ground in between the sessions and the later sessions were not preceded by habituation phases as we assumed the animals were already accustomed to the presence of the trays in the environment. See Supplementary Table S1 online for the dates of and treatments applied during each session.

The plots were visited daily during each session, between 9 am and sunset. From the second day of each session onwards, upon visiting each plot, we noted whether there were any signs of digging in the gravel and whether the camera trap recorded any visits of study species the previous night. If either of these occurred, we sifted through the gravel to count and record the number of food pellets remaining in the tray; a fresh 20 pellets were then mixed into the gravel. When there were no signs of foraging, we checked the condition of the food for signs of decay. If its condition was good—i.e. no signs of mould and the pellets were maintaining their form—we left the feeding trays without exchanging the food. Otherwise, we replaced the food in the trays every 2–4 days. We wore rubber gardening gloves whenever attending a station. Each day at each plot we transferred one fresh towel onto the bamboo cane using a clothes peg, taking care not to handle the towels directly to prevent contamination with other odours.

### Data analysis

The camera trap videos were uploaded to Trapper (www.os-conservation.org), a cloud application for managing data collected during camera trapping projects^42^. We used Trapper to classify the videos, recording the presence of species and the time animals spent doing different types of behaviour (see ethogram in Supplementary Table S2, and Supplementary Methods online). We then built an occupancy matrix using camtrapR to determine frequency of plot use of each of the mesocarnivores^47^. This determined the presence and absence of each mesocarnivore species at each plot each day (termed plot use hereinafter), which was considered to be from 14:00 on one afternoon till 14:00 the next. We picked this time as the cut-off as the study species are known to be largely inactive during the afternoon (e.g.^48,49^). GUDs were assigned to the species recorded foraging from a feeding tray the previous night. Eight GUDs were removed from the dataset as the camera traps had failed to trigger and it was impossible to establish the species foraging at the study plot. GUDs collected on days where more than one species visited (n = 29) were excluded from the dataset if both visiting species were filmed digging in the feeding tray (n = 8). To include data on patch avoidance, some previous studies recorded a maximum GUD value for days when animals did not visit, or each day that an animal visited but did not forage^14,16,50^. We decided against this because it would have imbalanced our dataset, causing it to be dominated by the highest value as animals did not forage or even visit on most nights at most plots. We carried out a separate analysis, a Chi-squared test, to determine whether mesocarnivores avoided foraging at plots exposed to wolf body odour: we compared between treatments the number of days a species visited study plots but did not forage with the number of days it visited *and* foraged.

To analyse the effects of risk on mesocarnivore foraging, we used an inference based modelling approach aimed at hypothesis testing, as described in Treddenick et al.^51^. We considered the GUDs as count data, as in Leo et al.^14^, and used a generalised linear mixed model (GLMM) with Poisson distribution to determine the effects of wolf encounter rates and wolf body odour treatment (and the interaction between them, to test for context dependence) on raccoon dog and fox GUDs. As covariates we included study period to control for the effect of seasonal variation in foraging behaviour, and the session day number (i.e. the day during the session that the GUD was collected) to control for the effect of habituation over the course of each session; plot number was included as a random factor to control for the effects of location. We used a GLMM with betabinomial distribution to test the effects of wolf encounter rates and wolf body odour (and the interaction between them) on plot use, which was grouped at the level of the session, as there were too many zeros in the dataset to run the model on the Bernoulli binomial dataset. The model included study period as a covariate, and plot number as a random factor for the same reasons as described above. To test the hypothesis that the landscape level risk context modifies the effect of wolf body odour treatment on GUDs and plot use, we used likelihood ratio tests to compare the full models described above with alternative, reduced models that did not include the interaction between these main effects. The interaction was a significant effect only in the raccoon dog GUD model (Supplementary Table S3 online); thus we chose the full model to be presented here. Whereas the fox GUD model, and the raccoon dog and fox plot use models without the interaction were more parsimonious (Supplementary Table S3 online), so in these cases we chose to present the reduced models without the interaction. Worth to mention is that the model diagnostics for the fox GUD model showed weak overdispersion (dispersion parameter = 0.407, p-value = 0.024, Supplementary Fig. S1 online); however, we believe this deviation was likely caused by limitations in the data (small sample size) used to fit the model, and not by erroneous model structure and thus we decided to present the results of this model here.

During preliminary analysis we also tested two further interactions that were removed from the final models to keep them as simple as possible. In the GUD models for both species, we included the interaction between wolf odour and day number to test whether mesocarnivores became habituated to the wolf odour over the course of sessions; this interaction was not significant. In both the GUD and plot use models, we also included the interaction between wolf odour and study period to test whether the mesocarnivore response to risk changed between study periods. The interaction between odour and study period was a significant predictor of raccoon dog GUDs; however, it was only significant because in study period 1 plots exposed to body odour had very high GUDs, which were likely an artefact caused by small sample size (see Supplementary Fig. S2 online). This interaction was not a significant predictor of fox GUDs (see Supplementary Fig. S3 online), nor of plot use in either species.

We also investigated whether the behaviour of mesocarnivores varied in response to wolf body odour and the landscape-scale gradient in wolf encounter rates. We attempted model the effects of the same variables as above on the proportions of time per visit spent doing different types of behaviour using binomial and betabinomial models (Supplementary Methods online). However, these models did not converge due to low sample sizes or had predicted responses with extremely wide confidence intervals. Thus we decided to omit this analysis from the paper.

Statistics were carried out in R v 4.0.3^52^ using the R package glmmTMB v 1.0.2.1^53^. Continuous variables in the models were scaled using the scale function in R. The Pearson correlation coefficients (r) between our two continuous variables, session day number and wolf encounter rates, were − 0.014 in the raccoon dog, and − 0.204 in the fox GUD datasets, indicating low levels of correlation. We evaluated the fit of models through visual inspection of standard model diagnostics plots using the package Dharma v 0.4.1^54^ (Supplementary Figs. S1, S4, S5, S6 online). For each statistical model, we used the predictorEffect function in the R package Effects v 4.2.0^55^ to calculate the marginal effects of each significant variable, which (when using its default settings) averages values of non-focal factors, weighting levels of factors in proportion to sample size.

## Results

We recorded 938 videos of target mesocarnivores, 642 videos of raccoon dogs, 174 of foxes, 90 of badgers and 32 of pine martens. Excluding three camera trap days on which the cameras failed, the study comprised 487 camera trap days in total, 243 days at control sites and 244 at treatment sites. Raccoon dogs visited on 125 camera trap days (25.7%), foxes on 66 (13.6%), badgers on 28 (5.75%) and pine martens on 18 (3.7%) (See also Supplementary Tables S1 and S4 online for data at the treatment and session level). A wolf visited only once. In total, we collected 83 usable GUDs (this number is lower than the number of days visited as mesocarnivores did not forage on each day that they visited): 54 GUDs for the raccoon dog, and 29 for the fox (See also Supplementary Table S4 online for data per treatment).

**Figure 3:**
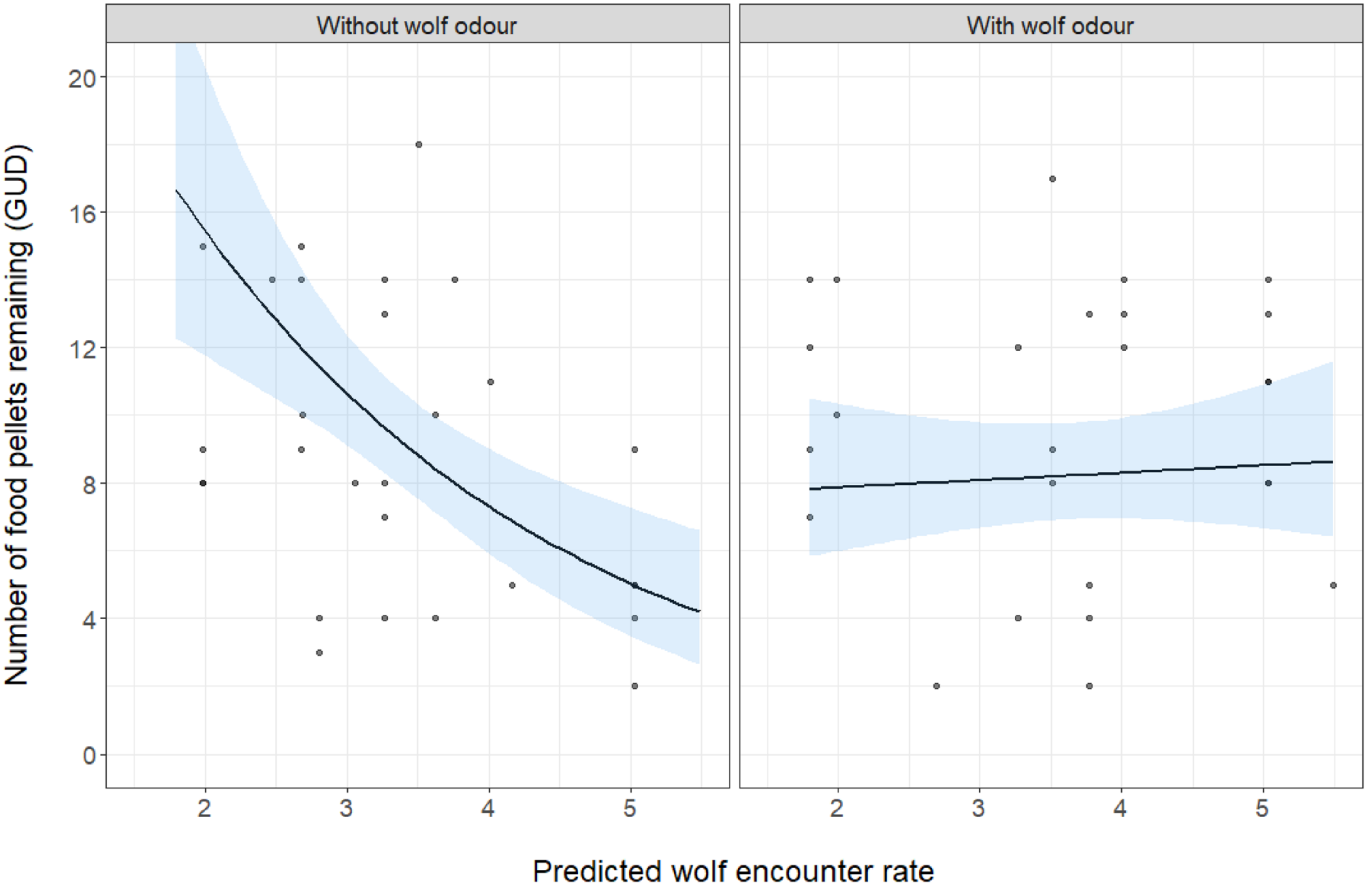
Predicted GUDs relative to wolf encounter rates for control and treatment plots based on the results of the Poisson GLMM. Ribbons represent the 95% confidence intervals around the predicted values. Points represent the raw GUDs. Wolf encounter rates refer to the pattern of space use of wolves in 500 × 500 m grid cells around the study plots (based on Bubnicki et al.^34^).

For raccoon dogs, wolf encounter rate, study period, session day number, and the interaction between landscape-scale wolf encounter rates and body odour treatment were significant predictors of GUDs (Table 1). At control plots, GUDs decreased (hence higher food depletion) with increasing landscape-scale wolf encounter rates, from 16 in areas with low wolf encounter rates to 4 in areas with high wolf encounter rates. At plots exposed to the wolf body odour treatment, there was no significant relationship between wolf risk and GUDs, with ca. 9 pieces left in trays both in areas of high and low risk (Fig. 3). Raccoon dog GUDs decreased substantially with each consecutive study period (Supplementary Fig. S7 online), and gradually with session day number (Supplementary Fig. S8 online). Fox GUDs did not vary with any of the tested predictor variables or covariates (Supplementary Table S5).

**Table 1:** Parameter estimates for the generalized linear mixed model with Poisson distribution describing raccoon dog giving up densities.

Parameters	Estimate	SE	Z	P
Intercept	2.609	0.139	18.821	< 0.001
Wolf encounter rate	− 0.406	0.104	− 3.889	**< 0.001**
Wolf body odour	− 0.083	0.113	− 0.734	0.463
Study period 2	− 0.396	0.126	− 3.132	**0.002**
Study period 3	− 0.955	0.190	− 5.040	**< 0.001**
Day of session	− 0.154	0.048	− 3.222	**0.001**
Wolf encounter rate × treatment	0.435	0.117	3.726	**< 0.001**

Significant values are in bold.

Raccoon dog and fox use of the study plots was unrelated to risk, and for both species study period 2 was a statistically significant predictor of higher plot use (Supplementary Table S6; Supplementary Figs. S9 and S10 online). Raccoon dogs and foxes did not appear to avoid foraging at wolf body odour plots, relative to control plots (χ^2^ = 0.317, P = 0.573; χ^2^ = 0.267, P = 0.605; respectively).

## Discussion

In contrast to the classical view that large carnivores primarily shape the behaviour of mesocarnivores through fear, recent studies have found that the facilitative effects can also be important^23,24^. Using a giving up density experiment we quantified the risk raccoon dogs and foxes perceive in response to wolf body odour across a landscape-scale gradient in wolf encounter rates in BF, an area with widespread large carnivore carrion provisioning. At locations with higher wolf encounter rates, raccoon dogs depleted feeding trays more (i.e. lower GUDs) than at plots with lower wolf encounter rates (i.e. ‘lower risk’ areas). Simulating immediate wolf presence by adding wolf body odour caused raccoon dogs to deplete feeding trays more (lower GUDs) at locations with low wolf encounter rates, but less (higher GUDs) at locations with high wolf encounter rates. These results suggest that in BF the suppressive effects of wolves play a subordinate role to their facilitative effects in determining raccoon dog and fox foraging behaviour. Wolf risk suppressed only raccoon dog foraging behaviour when wolf odour was present at locations with high wolf encounter rates, indicating a context-dependent risk effect. These findings provide further evidence that risk and reward can interactively shape the behaviour of mesocarnivores^23,24^ and call for a better understanding of how context modifies the ecological impacts of large carnivores.

If large carnivores suppressed their behaviour, we expected mesocarnivores to perceive higher foraging costs at study plots with wolf odour and/or high wolf encounter rates. However, the only support for the suppression hypothesis we found was the raccoon dog’s lower depletion of study plots (higher GUDs) with wolf body odour in areas with the highest wolf encounter rates. This result represents a context-dependent fear effect, whereby cues indicating immediate wolf presence (body odour) instil greater fear in raccoon dogs as wolf encounter rates increase. Except in this limited context, in line with our facilitation hypothesis, we found that wolf body odour and high wolf encounter rates did not increase the raccoon dog’s perceived costs of foraging. Raccoon dogs likely foraged at risky experimental foraging plots, as they do at large carnivore kill sites^7^, because they perceived the food benefits derived from these risky patches to outweigh the costs of the risk. However, it is not fully clear to us why raccoon dogs ate more dog pellets (i.e. perceived lower costs of foraging) in areas with high wolf encounter rates. One possibility is that in those areas chronic stress and heightened energy demands cause raccoon dogs to consume more carbohydrates than in safer areas^56^. Another possibility is that the personalities of raccoon dog individuals vary across the wolf risk gradient (as can occur in herbivores^8^), whereby areas with high wolf encounter rates are occupied by bolder individuals that are less sensitive to risk. We assume several raccoon dog individuals visited our plots, as the reported densities of raccoon dogs in BF are 1.7–5 per 10 km^2^^25^ and our study area in southeastern BF encompassed an area of ca. 60 km^2^. Also counterintuitively, exposing the raccoon dog to wolf body odour at plots in areas of low wolf encounter rates led to higher depletion of plots (lower GUDs) compared with control plots, indicating the wolf body odour somehow lowered the raccoon dog’s perceived costs of foraging in areas with low wolf encounter rates—but we are unable to provide a satisfactory explanation for this pattern based on the existing knowledge in the literature. Raccoon dog GUDs also decreased with session day number, suggesting they became somewhat habituated to our experimental plots the longer a session went on. Raccoon dogs depleted plots more (i.e. GUDs were lower) in late summer and autumn than in early summer, likely because their energy requirements increase over the year (e.g. increased food requirements as pups grow, or as they increase fat reserves in preparation for winter) and the availability of alternative food sources varies. Also in line with our facilitation hypothesis, the GUD results suggest that fox foraging costs did not vary with experimentally added wolf body odour, wolf encounter rates or the interaction between them, again suggesting that in BF large carnivore risk is less important than facilitation in shaping fox foraging behaviour. The fox’s foraging costs also did not vary with day of session, suggesting foxes did not habituate to the experimental plots over the course of sessions. In contrast to the raccoon dog, fox foraging costs did not vary with study period, and this may be because the fox has sufficient alternative food sources available during these different times of the year to satisfy any changes in energy requirements. As tolerance towards risk depends on the physiological state of the animal^12^, which varies over the year, a higher tolerance to risk would be expected in the periods of highest energy requirements. However, similarly to the raccoon dog, study period did not modify the fox’s response to wolf body odour (Supplementary Figs. S2 and S3 online). This result reinforces the main message—that suppression via large carnivore-induced risk effects plays a subordinate role to facilitative effects in determining these mesocarnivores’ foraging behaviour in this system. Previous GUD studies have found the fox to respond both positively and negatively to large carnivore risk cues^14,16,50^, and we discuss why the mesocarnivore response to risk is so varied below.

If large carnivores supressed mesocarnivore behaviour we expected mesocarnivores to show lower use of study plots with wolf odour and/or higher wolf encounter rates. However, in accordance with our facilitation hypothesis, their use of experimental foraging patches did not vary with wolf body odour or wolf encounter rates. The lack of response to wolf encounter rates contrasts with our previous study on badgers in this system that showed that their use of setts decreased with increasing wolf risk across the same landscape-scale wolf encounter risk gradient^57^. However, differences in their ecology may explain why badgers respond differently to landscape level wolf risk than raccoon dogs and foxes, e.g. badgers are much less dependent on wolf kills, with ungulate carcasses forming only 0.3% of biomass in the badger diet^25^. The different response variables studied in these studies may also explain these differences (foraging patch use vs. sett use). The lack of avoidance of plots exposed to body odour could also be explained by mesocarnivores repeatedly approaching the scents to acquire ‘new’ information about large carnivores^22^. Animals are known to investigate scents for several reasons, e.g. to evaluate predator presence and determine the true risk of predation: this does not necessarily mean that a risk cue has no effect as the deterrent may still be ‘working’ if the animal retreats without consumption^22^. However, in our study mesocarnivores did not forage less often (i.e. avoid foraging) at plots exposed to wolf body odour, suggesting the wolf body odour cue simply did not have a deterrent effect on foraging patch use. On the other hand, the lack of attraction to risky study plots contrasts with previous studies showing raccoon dogs and foxes track wolves to find their kills^7,25^. This difference could be related to differences in the reward as ungulate carcasses may be a more valuable resource than dog pellets. Still, the lack of avoidance of risky plots shows that even food subsidies in the form of dog pellets are enough to draw mesocarnivores to risky plots, and therefore suggest suppression is less important than facilitation in determine foraging patch choice in BF. Both species used the plots more often in late summer compared with early summer likely due to seasonal changes in their behaviour^25^ related to pup-rearing, higher abundances of mesocarnivores in late summer due to sub-adults dispersing from their parents, and seasonal changes in the availability of alternative food sources.

Similarly to recent studies carried out in North American systems^23,24^, our results suggest that large carnivore-induced risk is not a primary driver of mesocarnivore behaviour. We do not believe the general lack of observable negative response to risk was due to limitations in our study design. The quantities of wolf odour added at our plots should have been sufficient to simulate the repeated visit of a wolf to the area, as we left the towels in the resting area of a captive wolf for three weeks, ensuring they were imbued with wolf odour. Still, it could be argued that only adding body odour is insufficient to simulate real wolf presence and that mesocarnivores somehow ‘knew’ there was no real wolf present at the plots. But in contrast to other olfactory cues such as scats and urine, body odour has been argued to provide the most direct simulation of actual carnivore presence and has been shown to be effective in instilling fear in prey^14,38,39^. A limitation of our study is that we used the odour from only one wolf, from outside the study area; however, we are not aware of any knowledge that indicates mesocarnivores can distinguish between and perceive varying levels of risk in response to the odour of different individuals of a large carnivore species, or that they find non-resident predators less scary than residents. Yet future studies may wish to consider sourcing odour from both sexes, more than one individual, members of a pack, or from wild wolves. It is noteworthy that we mainly carried out our field studies during the warm season, when alternative food sources are abundant. Animals are predicted to be at their most risk-tolerant when they are hungry (see^12^). That we did not observe behavioural suppression (in either response variable) even in the season when fear effects should be strongest indicates that in this study area suppression is a far less important determinant of mesocarnivore foraging behaviour than facilitation.

Mesocarnivore foraging strategies have been found to vary from risk averse to risk tolerant: studies have shown mesocarnivores avoid and reduce their foraging at sites with predator cues present^14,16^, while others have shown they are attracted to large carnivores and their kills^23,24^ and can even increase their foraging in the presence of wolf scats^50^. These varying responses raise the question of which contextual factors in these study areas cause a strategy based on risk-avoidance to switch to one based on risk-tolerance? In their study, Sivy et al.^23^ suggested that high large carnivore densities may be an important factor, as they may increase scavenging benefits for mesocarnivores, making avoidance of large carnivores a disadvantageous, costly strategy. In this context, mesocarnivores deliberately seek out risky places to forage as the peaks in the landscapes of fear and food overlap. In line with the risk-allocation hypothesis, we predict that in areas with dense populations of large carnivores such as BF, with widespread food subsidies in the form of large carnivore kills, mesocarnivores are likely to experience chronically high risk while foraging. This may cause them to become risk-tolerant and put less effort into antipredator behaviour^58^. In such areas, mesocarnivores likely need a variety or combination of cues (olfactory, visual, auditory), or even the live presence of a large carnivore before they take risk avoidance measures^59,60^. Notably, our study also shows that the mesocarnivore response can vary as the context changes within an area, as has been shown before in ungulates^61,62^. It suggests mesocarnivores are capable of changing their sensitivity to risk cues when moving from low to high risk areas. This weighing of risk and reward presumably allows them to choose the most optimal foraging strategy ‘on the fly’ as they navigate around the peaks and troughs of a risk landscape.

To conclude, our study is the first in a European mesocarnivore community to suggest that the effects of large carnivore risk on mesocarnivore behaviour can be outweighed by facilitative effects. This knowledge will help us understand the ecological effects of the ongoing large carnivore recolonisation of Europe^63^. Our study and previous studies show that contextual variation can modify how risk effects shape prey behaviour (see^10,12,21^). Yet, it remains unclear which contextual factors determine whether suppression or facilitation dominates the interactions between large- and meso-carnivores. Most studies have focussed on testing the effect of risk cues (e.g. odour, scat, predator presence) on prey behaviour, but have not considered how the prey response to risk depends on the context. We suggest that rather than testing the effects of single risk cues on prey behaviour, future studies should focus on understanding how context modifies the ecological impacts of large carnivores.

## Supplementary Information

Supplementary Information.

## Data Availability

The datasets used and/or analysed during the current study are available from the corresponding author on reasonable request.

## References

[1] Elmhagen B, Rushton SP (2007). Trophic control of mesopredators in terrestrial ecosystems: Top-down or bottom-up?. Ecol. Lett..

[2] Newsome TM (2017). Top predators constrain mesopredator distributions. Nat. Commun..

[3] Prugh LR, Sivy KJ (2020). Enemies with benefits: Integrating positive and negative interactions among terrestrial carnivores. Ecol. Lett..

[4] Lima SL, Dill LM (1990). Behavioral decisions made under the risk of predation: A review and prospectus. Can. J. Zool..

[5] Suraci JP, Clinchy M, Dill LM, Roberts D, Zanette LY (2016). Fear of large carnivores causes a trophic cascade. Nat. Commun..

[6] Suraci JP, Clinchy M, Zanette LY, Wilmers CC (2019). Fear of humans as apex predators has landscape-scale impacts from mountain lions to mice. Ecol. Lett..

[7] Selva N, Jȩdrzejewska B, Jȩdrzejewski W, Wajrak A (2005). Factors affecting carcass use by a guild of scavengers in European temperate woodland. Can. J. Zool..

[8] McArthur C, Banks PB, Boonstra R, Forbey JS (2014). The dilemma of foraging herbivores: Dealing with food and fear. Oecologia.

[9] Ripple WJ (2014). Status and ecological effects of the world’s largest carnivores. Science.

[10] Kuijper DPJ (2016). Paws without claws? Ecological effects of large carnivores in anthropogenic landscapes. Proc. R. Soc. B Biol. Sci..

[11] Laundré JW, Hernández L, Altendorf KB (2001). Wolfes, elk, and bison: Reestablishing the ‘landscape of fear’ in Yellowstone National Park, U.S.A. Can. J. Zool..

[12] Gaynor KM, Brown JS, Middleton AD, Power ME, Brashares JS (2019). Landscapes of fear: Spatial patterns of risk perception and response. Trends Ecol. Evol..

[13] Ritchie EG, Johnson CN (2009). Predator interactions, mesopredator release and biodiversity conservation. Ecol. Lett..

[14] Leo V, Reading RP, Letnic M (2015). Interference competition: Odours of an apex predator and conspecifics influence resource acquisition by red foxes. Oecologia.

[15] Clinchy M (2016). Fear of the human “super predator” far exceeds the fear of large carnivores in a model mesocarnivore. Behav. Ecol..

[16] Haswell PM, Jones KA, Kusak J, Hayward MW (2018). Fear, foraging and olfaction: how mesopredators avoid costly interactions with apex predators. Oecologia.

[17] Switalski TA (2003). Coyote foraging ecology and vigilance in response to gray wolf reintroduction in Yellowstone National Park. Can. J. Zool..

[18] Wikenros C, Jarnemo A, Frisén M, Kuijper DPJ, Schmidt K (2017). Mesopredator behavioral response to olfactory signals of an apex predator. J. Ethol..

[19] Palomares F, Ferreras P, Fedriani JM, Delibes M (1996). Spatial relationships between Iberian Lynx and other carnivores in an area of south-western Spain. J. Appl. Ecol..

[20] Salo P, Nordström M, Thomson RL, Korpimäki E (2008). Risk induced by a native top predator reduces alien mink movements. J. Anim. Ecol..

[21] Haswell PM, Kusak J, Hayward MW (2017). Large carnivore impacts are context-dependent. Food Webs.

[22] Parsons MH (2018). Biologically meaningful scents: A framework for understanding predator–prey research across disciplines. Biol. Rev..

[23] Sivy KJ, Pozzanghera CB, Grace JB, Prugh LR (2017). Fatal attraction? Intraguild facilitation and suppression among predators. Am. Nat..

[24] Ruprecht J (2021). Variable strategies to solve risk-reward tradeoffs in carnivore communities. Proc. Natl. Acad. Sci. USA..

[25] Jędrzejewska B, Jędrzejewski W (1998). Predation in Vertebrate Communities.

[26] Jȩdrzejewski W (2002). Kill rates and predation by wolves on ungulate populations in Białowieża primeval forest (Poland). Ecology.

[27] Selva, N. The role of scavenging in the predator community of Białowieża Primeval Forest (Poland). *PhD Thesis*. (University of Sevilla, 2004).

[28] Kowalczyk R, Zalewski A, Jędrzejewska B, Ansorge H, Bunevich AN (2009). Reproduction and mortality of invasive raccoon dogs (Nyctereutes procyonoides) in the Biatowieža Primeval Forest (eastern Poland). Ann. Zool. Fennici.

[29] Ballard WB, Carbyn LN, Smith DW, Mech D, Boitani L (2003). Wolf interactions with non-prey. Wolves: Behavior, Ecology, and Conservation.

[30] Brown JS (1988). Patch use as an indicator of habitat preference, predation risk, and competition. Behav. Ecol. Sociobiol..

[31] Bedoya-Perez MA, Carthey AJR, Mella VSA, McArthur C, Banks PB (2013). A practical guide to avoid giving up on giving-up densities. Behav. Ecol. Sociobiol..

[32] Kwiatkowski W (1994). Vegetation landscapes of Białowieża Forest. Phytocoen. Suppl. Cart. Geobot.

[33] European Court of Justice Judgment of the Court (Grand Chamber) of 17 April 2018. European Commission vs. Republic of Poland. Case C-441/17. https://curia.europa.eu/jcms/upload/docs/application/pdf/2018-04/cp180048en.pdf.

[34] Bubnicki JW, Churski M, Schmidt K, Diserens TA, Kuijper DPJ (2019). Linking spatial patterns of terrestrial herbivore community structure to trophic interactions. Elife.

[35] Kowalczyk R, Bunevich AN, Jędrzejewska B (2000). Badger density and distribution of setts in Bialowieza Primeval Forest (Poland and Belarus) compared to other Eurasian populations. Acta Theriol..

[36] Jędrzejewski W, Schmidt K, Theuerkauf J, Jędrzejewska B, Kowalczyk R (2007). Territory size of wolves *Canis lupus*: Linking local (Białowieża Primeval Forest, Poland) and holarctic-scale patterns. Ecography.

[37] Schmidt K, Jędrzejewski W, Okarma H, Kowalczyk R (2009). Spatial interactions between grey wolves and Eurasian lynx in Białowieża Primeval Forest, Poland. Ecol. Res..

[38] Bytheway JP, Carthey AJR, Banks PB (2013). Risk vs reward: How predators and prey respond to aging olfactory cues. Behav. Ecol. Sociobiol..

[39] Carthey AJR, Banks PB (2016). Naiveté is not forever: responses of a vulnerable native rodent to its long term alien predators. Oikos.

[40] Blanchard CD, Blanchard RJ, Whishaw IQ, Kolb B (2004). Antipredator DEFENSE. The Behavior of the Laboratory Rat: A Handbook with Tests.

[41] Masini CV, Sauer S, Campeau S (2005). Ferret odor as a processive stress model in rats: Neurochemical, behavioral, and endocrine evidence. Behav. Neurosci..

[42] Bubnicki JW, Churski M, Kuijper DPJ (2016). Trapper: An open source web-based application to manage camera trapping projects. Methods Ecol. Evol..

[43] Jędrzejewski W, Schmidt K, Theuerkauf J, Jędrzejewska B, Okarma H (2001). Daily movements and territory use by radio-collared wolves (*Canis lupus*) in Bialowieza Primeval Forest in Poland. Can. J. Zool..

[44] Theuerkauf J, Jędrzejewski W, Schmidt K, Gula R (2003). Spatiotemporal segregation of wolves from humans in the Bialowieza Forest (Poland). J. Wildl. Manage..

[45] Theuerkauf J, Rouys S, Jędrzejewski W (2003). Selection of den, rendezvous, and resting sites by wolves in the Bialowieza Forest, Poland. Can. J. Zool..

[46] Miller BJ, Harlow HJ, Harlow TS, Biggins D, Ripple WJ (2012). Trophic cascades linking wolves (*Canis lupus*), coyotes (*Canis latrans*), and small mammals. Can. J. Zool..

[47] Niedballa J, Sollmann R, Courtiol A, Wilting A (2016). camtrapR: An R package for efficient camera trap data management. Methods Ecol. Evol..

[48] Zoller H, Drygala F (2013). Activity patterns of the invasive raccoon dog (Nyctereutes procyonoides) in North East Germany. Folia Zool..

[49] Díaz-Ruiz F, Caro J, Delibes-Mateos M, Arroyo B, Ferreras P (2016). Drivers of red fox (*Vulpes vulpes*) daily activity: Prey availability, human disturbance or habitat structure?. J. Zool..

[50] Mukherjee S, Zelcer M, Kotler BP (2009). Patch use in time and space for a meso-predator in a risky world. Oecologia.

[51] Tredennick AT, Hooker G, Ellner SP, Adler PB (2021). A practical guide to selecting models for exploration, inference, and prediction in ecology. Ecology.

[52] Team, R. C. *R: A Language and Environment for Statistical Computing*. (R Foundation for Statistical Computing, 2021).

[53] Magnusson, A. *et al*. *R Package ‘glmmTMB’*. (2020).

[54] Hartig, F. *R Package ‘DHARMa: Residual Diagnostics for Hierarchical (Multi-level/Mixed) Regression Models’* (2021).

[55] Fox, J. *et al*. *R Package ‘effects’*. (2020).

[56] Hawlena D, Schmitz OJ (2010). Physiological stress as a fundamental mechanism linking predation to ecosystem functioning. Am. Nat..

[57] Diserens TA (2021). Fossoriality in a risky landscape: Badger sett use varies with perceived wolf risk. J. Zool..

[58] Lima SL, Bednekoff PA (1999). Temporal variation in danger drives antipredator behavior: The predation risk allocation hypothesis. Am. Nat..

[59] Scheinin S, Yom-Tov Y, Motro U, Geffen E (2006). Behavioural responses of red foxes to an increase in the presence of golden jackals: A field experiment. Anim. Behav..

[60] Vanak AT, Thaker M, Gompper ME (2009). Experimental examination of behavioural interactions between free-ranging wild and domestic canids. Behav. Ecol. Sociobiol..

[61] Creel S, Winnie JA, Christianson D, Liley S (2008). Time and space in general models of antipredator response: Tests with wolves and elk. Anim. Behav..

[62] Dröge E, Creel S, Becker MS, M’soka J (2017). Risky times and risky places interact to affect prey behaviour. Nat. Ecol. Evol..

[63] Chapron G (2014). Recovery of large carnivores in Europe’s modern human-dominated landscapes. Science.

