# Supplementary material for: Wolf risk fails to inspire fear in two mesocarnivores suggesting facilitation prevails
Source: Sci Rep. 2022 Oct 1;12:16469. doi: 10.1038/s41598-022-20725-3 (PMC9526698; doi:10.1038/s41598-022-20725-3)
Supplement: Supplementary file 1 — Supplementary Information. [file 41598_2022_20725_MOESM1_ESM.docx]

**Electronic supplementary material**

**Wolf risk fails to inspire fear in two mesocarnivores suggesting facilitation prevails**

Tom A. Diserens^1,2,*^, Marcin Churski^1^, Jakub W. Bubnicki^1^, Andrzej Zalewski^1^, Marcin Brzezinski^2^, Dries P.J. Kuijper^1^

^1^Mammal Research Institute, Polish Academy of Sciences, ul. Stoczek 1, 17-230 Białowieża.

^2^Faculty of Biology, University of Warsaw, ul. Miecznikowa, 1, 02‑097 Warsaw

*Corresponding author: tdiserens@ibs.bialowieza.pl

**Supplementary Table S1** Data for each study period and session, with a list of study plots and the treatment they were subject to. The numbers in the species columns refer to the number of days during each session on which a species was recorded.

| Study period | Session | Study Plot | Treatment | Start date | End date | Length (# days) | Raccoon dog (# days) | Fox  (# days) | Badger  (# days) | Pine marten  (# days) |
| --- | --- | --- | --- | --- | --- | --- | --- | --- | --- | --- |
| 1 | 1 | 2 | Wolf scent | 20/06/2019 | 30/06/2019 | 10 | 1 | 2 | 0 | 0 |
| 1 | 1 | 3 | Wolf scent | 20/06/2019 | 30/06/2019 | 10 | 1 | 0 | 0 | 0 |
| 1 | 1 | 7 | No scent | 20/06/2019 | 30/06/2019 | 10 | 5 | 0 | 0 | 0 |
| 1 | 1 | 9 | No scent | 20/06/2019 | 30/06/2019 | 10 | 0 | 0 | 0 | 0 |
| 1 | 1 | 10 | No scent | 20/06/2019 | 30/06/2019 | 10 | 4 | 2 | 0 | 0 |
| 1 | 1 | 14 | Wolf scent | 20/06/2019 | 30/06/2019 | 10 | 10 | 1 | 0 | 0 |
| 1 | 1 | 15 | Wolf scent | 20/06/2019 | 30/06/2019 | 10 | 0 | 6 | 0 | 0 |
| 1 | 1 | 18 | No scent | 20/06/2019 | 30/06/2019 | 10 | 0 | 0 | 0 | 0 |
| 1 | 1 | 27 | No scent | 20/06/2019 | 30/06/2019 | 10 | 2 | 2 | 0 | 0 |
| 1 | 1 | 29 | Wolf scent | 20/06/2019 | 30/06/2019 | 10 | 6 | 0 | 0 | 0 |
| 1 | 2 | 1 | No scent | 03/07/2019 | 14/07/2019 | 11 | 4 | 3 | 0 | 0 |
| 1 | 2 | 5 | Wolf scent | 03/07/2019 | 14/07/2019 | 11 | 0 | 1 | 0 | 0 |
| 1 | 2 | 8 | Wolf scent | 03/07/2019 | 14/07/2019 | 11 | 5 | 0 | 2 | 0 |
| 1 | 2 | 11 | Wolf scent | 03/07/2019 | 14/07/2019 | 11 | 0 | 0 | 0 | 0 |
| 1 | 2 | 12 | No scent | 03/07/2019 | 14/07/2019 | 11 | 5 | 0 | 0 | 0 |
| 1 | 2 | 16 | No scent | 03/07/2019 | 14/07/2019 | 11 | 0 | 0 | 0 | 0 |
| 1 | 2 | 23 | No scent | 03/07/2019 | 14/07/2019 | 11 | 1 | 5 | 0 | 0 |
| 1 | 2 | 25 | Wolf scent | 03/07/2019 | 14/07/2019 | 11 | 0 | 0 | 0 | 0 |
| 1 | 2 | 26 | No scent | 03/07/2019 | 14/07/2019 | 11 | 0 | 0 | 0 | 0 |
| 1 | 2 | 28 | Wolf scent | 03/07/2019 | 14/07/2019 | 11 | 23 | 0 | 2 | 0 |
| 2 | 3 | 1 | No scent | 11/08/2019 | 19/08/2019 | 8 | 2 | 4 | 0 | 1 |
| 2 | 3 | 5 | No scent | 11/08/2019 | 19/08/2019 | 8 | 1 | 1 | 0 | 0 |
| 2 | 3 | 7 | Wolf scent | 11/08/2019 | 19/08/2019 | 8 | 9 | 0 | 0 | 0 |
| 2 | 3 | 8 | No scent | 11/08/2019 | 19/08/2019 | 8 | 11 | 0 | 2 | 0 |
| 2 | 3 | 9 | Wolf scent | 11/08/2019 | 19/08/2019 | 8 | 12 | 0 | 1 | 0 |
| 2 | 3 | 10 | Wolf scent | 11/08/2019 | 19/08/2019 | 8 | 34 | 2 | 0 | 0 |
| 2 | 3 | 11 | No scent | 11/08/2019 | 19/08/2019 | 8 | 3 | 1 | 0 | 1 |
| 2 | 3 | 12 | Wolf scent | 11/08/2019 | 19/08/2019 | 8 | 0 | 0 | 0 | 0 |
| 2 | 3 | 15 | No scent | 11/08/2019 | 19/08/2019 | 8 | 7 | 3 | 0 | 1 |
| 2 | 3 | 27 | Wolf scent | 11/08/2019 | 19/08/2019 | 8 | 4 | 2 | 1 | 0 |
| 2 | 4 | 2 | Wolf scent | 15/09/2019 | 22/09/2019 | 7 | 3 | 5 | 0 | 0 |
| 2 | 4 | 3 | No scent | 15/09/2019 | 22/09/2019 | 7 | 5 | 1 | 0 | 0 |
| 2 | 4 | 14 | No scent | 15/09/2019 | 22/09/2019 | 7 | 21 | 3 | 2 | 4 |
| 2 | 4 | 16 | Wolf scent | 15/09/2019 | 22/09/2019 | 7 | 1 | 5 | 1 | 0 |
| 2 | 4 | 18 | No scent | 15/09/2019 | 22/09/2019 | 7 | 4 | 0 | 4 | 0 |
| 2 | 4 | 23 | Wolf scent | 15/09/2019 | 22/09/2019 | 7 | 5 | 1 | 0 | 0 |
| 2 | 4 | 25 | No scent | 15/09/2019 | 22/09/2019 | 7 | 2 | 2 | 3 | 1 |
| 2 | 4 | 26 | Wolf scent | 15/09/2019 | 22/09/2019 | 7 | 0 | 1 | 0 | 1 |
| 2 | 4 | 28 | Wolf scent | 15/09/2019 | 22/09/2019 | 7 | 1 | 3 | 1 | 2 |
| 2 | 4 | 29 | No scent | 15/09/2019 | 22/09/2019 | 7 | 15 | 0 | 0 | 2 |
| 3 | 5 | 1 | Wolf scent | 08/10/2019 | 16/10/2019 | 8 | 4 | 5 | 0 | 0 |
| 3 | 5 | 5 | Wolf scent | 08/10/2019 | 16/10/2019 | 8 | 1 | 0 | 0 | 0 |
| 3 | 5 | 8 | Wolf scent | 08/10/2019 | 16/10/2019 | 8 | 0 | 0 | 6 | 0 |
| 3 | 5 | 10 | No scent | 08/10/2019 | 16/10/2019 | 8 | 1 | 0 | 0 | 0 |
| 3 | 5 | 11 | Wolf scent | 08/10/2019 | 16/10/2019 | 8 | 1 | 2 | 1 | 0 |
| 3 | 5 | 12 | No scent | 08/10/2019 | 16/10/2019 | 8 | 0 | 0 | 0 | 1 |
| 3 | 5 | 15 | Wolf scent | 08/10/2019 | 16/10/2019 | 8 | 0 | 2 | 0 | 0 |
| 3 | 5 | 26 | No scent | 08/10/2019 | 16/10/2019 | 8 | 4 | 0 | 0 | 0 |
| 3 | 5 | 27 | No scent | 08/10/2019 | 16/10/2019 | 8 | 1 | 2 | 1 | 1 |
| 3 | 5 | 28 | No scent | 08/10/2019 | 16/10/2019 | 8 | 11 | 0 | 3 | 0 |
| 3 | 6 | 2 | No scent | 26/10/2019 | 31/10/2019 | 5 | 0 | 2 | 0 | 2 |
| 3 | 6 | 3 | Wolf scent | 26/10/2019 | 31/10/2019 | 5 | 0 | 0 | 0 | 0 |
| 3 | 6 | 7 | No scent | 26/10/2019 | 31/10/2019 | 5 | 0 | 1 | 0 | 0 |
| 3 | 6 | 8 | No scent | 26/10/2019 | 31/10/2019 | 5 | 0 | 0 | 2 | 1 |
| 3 | 6 | 14 | Wolf scent | 26/10/2019 | 31/10/2019 | 5 | 4 | 1 | 0 | 0 |
| 3 | 6 | 16 | No scent | 26/10/2019 | 31/10/2019 | 5 | 0 | 0 | 1 | 0 |
| 3 | 6 | 18 | Wolf scent | 26/10/2019 | 31/10/2019 | 5 | 0 | 0 | 1 | 0 |
| 3 | 6 | 23 | No scent | 26/10/2019 | 31/10/2019 | 5 | 0 | 0 | 0 | 0 |
| 3 | 6 | 25 | Wolf scent | 26/10/2019 | 31/10/2019 | 5 | 0 | 0 | 0 | 0 |
| 3 | 6 | 29 | Wolf scent | 26/10/2019 | 31/10/2019 | 5 | 0 | 0 | 0 | 1 |

**Supplementary Table S2** Ethogram used to classify the behaviour of mesocarnivores recorded on camera trap videos during the giving up densities field study. For the final analysis, similar behaviour types were combined (movement and vigilance were not combined with any other behaviour types).

| **Behaviour** | **Description** | **May be accompanied by** | **Combined as** |
| --- | --- | --- | --- |
| Sniffing air or ground | Nose directed at ground or forest canopy. | Movement. | Movement |
| Movement | Moving with determination, i.e. not sniffing. | Head movement, looking around |  |
| Sniffing or interacting with feeding tray | Eyes and nose directed at bucket or its vicinity. Bucket sniffing event ends when animal begins to dig or masticate. | Scratching, biting or licking the tray. | Foraging |
| Foraging | Ether digging or masticating in or around the bucket, sometimes interspersed with bouts of sniffing. | Movement, looking around while masticating |  |
| Sniffing or interacting with towel or bamboo stick | Animal’s eyes and nose directed at the towel or stick. | Movement | n/a |
| Vigilance | Head is level with or above the shoulders, but eyes facing below the forest canopy. Animal is not looking at the bucket, towel or ground. Animal is not doing anything else. | Pricked ears. Up and down movement of the head. Looking at the camera. | n/a |

**Supplementary Table S3** Results of maximum likelihood ratio tests testing the hypotheses that the landscape level risk context modifies the effect of the wolf body odour treatment on GUDs and plot use. We compared the full models that included the interaction between these main effects with alternative, reduced models without the interaction.

| **Type of model** | Df | AIC | Chisq | P |
| --- | --- | --- | --- | --- |
| **Raccoon dog GUDs** |  |  |  |  |
| Wolf encounter rates * wolf body odour + study period + day of session | 8 | 292.88 | 14.34 | <0.001 |
| Wolf encounter rates + wolf body odour + study period + day of session | 7 | 305.22 |  |  |
| **Raccoon dog plot use** |  |  |  |  |
| Wolf encounter rates * wolf body odour + study period | 8 | 240.48 | 0.192 | 0.661 |
| Wolf encounter rates + wolf body odour + study period | 7 | 221.91 |  |  |
| **Fox GUDs** |  |  |  |  |
| Wolf encounter rates * wolf body odour + study period + day of session | 8 | 153.47 | 0.173 | 0.677 |
| Wolf encounter rates + wolf body odour + study period + day of session | 7 | 151.65 |  |  |
| **Fox plot use** |  |  |  |  |
| Wolf encounter rates * wolf body odour + study period | 8 | 171.99 | 0.438 | 0.508 |
| Wolf encounter rates + wolf body odour + study period | 7 | 170.43 |  |  |

**Supplementary Table S4** Raw data detailing for each study species the number of days visited, days with/without foraging, and days GUDs could be determined. GUDs were not determinable for all days animals foraged, due to scenarios such as two animals foraging, or rain having disintegrated the dog pellets. Days refer to camera trap days.

|  | Plots without wolf scent | | | | Plots with wolf scent | | | | | | | Total for both treatments | | | |
| --- | --- | --- | --- | --- | --- | --- | --- | --- | --- | --- | --- | --- | --- | --- | --- |
| Species | Visited  (# days) | Visited without foraging (# days) | Foraged  (# days) | Usable GUDs (#) | |  | Visited  (# days) | Visited without foraging (# days) | Foraged  (# days) | Usable GUDs (#) |  | Visited  (#  days) | Visited without foraging (# days) | Foraged  (# days) | Usable GUDs (#) |
| Raccoon dog | 66 | 34 | 32 | 28 | |  | 58 | 26 | 32 | 26 |  | 124 | 60 | 64 | 54 |
| Fox | 32 | 13 | 19 | 14 | |  | 34 | 17 | 17 | 15 |  | 66 | 30 | 36 | 29 |
| Badger | 14 | 6 | 8 | 6 | |  | 14 | 7 | 7 | 6 |  | 28 | 13 | 15 | 12 |
| Pine marten | 14 | 11 | 4 | 1 | |  | 4 | 4 | 0 | 0 |  | 18 | 14 | 4 | 1 |

**Supplementary Table S5** Parameter estimates for the generalized linear mixed model with Poisson distribution describing fox giving up densities.

| **Parameters** | **Estimate** | **SE** | **Z** | **P** |
| --- | --- | --- | --- | --- |
| Intercept | 2.509 | 0.096 | 26.140 | **< 0.001** |
| Wolf encounter rate | 0.008 | 0.060 | 0.130 | **0.896** |
| Wolf body odour | -0.034 | 0.125 | -0.276 | **0.783** |
| Study period 2 | 0.056 | 0.134 | 0.415 | **0.678** |
| Study period 3 | -0.066 | 0.154 | -0.431 | **0.667** |
| Day of session | -0.036 | 0.065 | -0.555 | **0.579** |

**Supplementary Table S6** Parameter estimates for the generalized linear mixed model with beta-binomial distribution describing raccoon dog and fox use of study plots per session (a binomial response variable representing daily presence and absence of each species at study plots during each session).

| **Parameters** | **Estimate** | **SE** | **Z** | **P** |
| --- | --- | --- | --- | --- |
| **Raccoon dog** |  |  |  |  |
| Intercept | -1.400 | 0.377 | -3.714 | <0.001 |
| Wolf odour treatment | 0.256 | 0.209 | 1.222 | 0.222 |
| Wolf encounter rate | -0.244 | 0.364 | -0.670 | 0.503 |
| Study period 2 | 1.250 | 0.415 | 3.012 | 0.003 |
| Study period 3 | -0.434 | 0.488 | -0.891 | 0.373 |
| **Fox** |  |  |  |  |
| Intercept | -2.730 | 0.478 | -5.716 | <0.001 |
| Wolf odour treatment | 0.319 | 0.274 | 1.164 | 0.244 |
| Wolf encounter rate | 0.107 | 0.369 | 0.291 | 0.771 |
| Study period 2 | 1.142 | 0.429 | 2.661 | 0.008 |
| Study period 3 | 0.172 | 0.491 | 0.350 | 0.726 |

**Supplementary Methods** for analysing mesocarnivore behavioural data recorded by camera traps.

In line with the ethogram (Supplementary Table 2) we scored the behaviour of animals by recording the number of seconds during each clip that the animal spent doing the following behaviours: vigilance, movement, ground sniffing, bucket sniffing, foraging, interacting with towel, and unclassified. We treated the behaviour types as mutually exclusive. Behaviour was only scored when i) the animal’s head was visible within the camera’s field of view, ii) when the behaviour was clearly discernible, which discounted scenarios such as when the animal was too far away from the camera to be seen clearly, and iii) when the behaviour type fitted into one of our categories, which discounted scenarios with rare types of behaviour, such as grooming or interacting with other individuals. When the animal was visible but did not satisfy these criteria, the behaviour was marked as ‘unclassified’. If there were more than two individuals in the field of view, only the behaviour of the individual nearest the bucket was scored. If neither foraged, the one whose behaviour was visible for the longest was selected. This criteria was chosen because scoring the behaviour of both individuals may have led to the inclusion of spurious data due to one individual affecting the behaviour of the other, e.g. by blocking its entry to the foraging bucket. On 29 videos (usually 1 min long) there were more than 1 raccoon dogs on a video. Any time they spent interacting with each other was classed as ‘unclassified behaviour’. On one occasion two species were present together – and the same rule was applied. Later, to simplify the analysis, we combined similar types of behaviour into four more general behavioural categories: vigilance, movement, towel interaction, and foraging. New visits were considered to begin if the period between two videos was greater than 15 min. The number of seconds spent carrying out each behaviour type during each visit was summed for all the videos corresponding to that visit. We calculated the proportion of time spent doing each behaviour during each visit (behaviour duration/total time behaviour was visible), making four response variables per species. The data also gave us the visit duration, which we also attempted to model. The binomial and beta binomial models built upon these datasets to predict mesocarnivore behaviour did not convergence due to low sample size or had predicted responses with extremely wide confidence intervals. Thus we excluded this data from the main part of the study.

**Supplementary Figure S1** Fox GUD model diagnostic plots. Note the significant dispersion test. We believe this deviation was likely caused by limitations in the data (small sample size) used to fit the model, and not by erroneous model structure and thus we decided to present the results of this model in the manuscript.

**
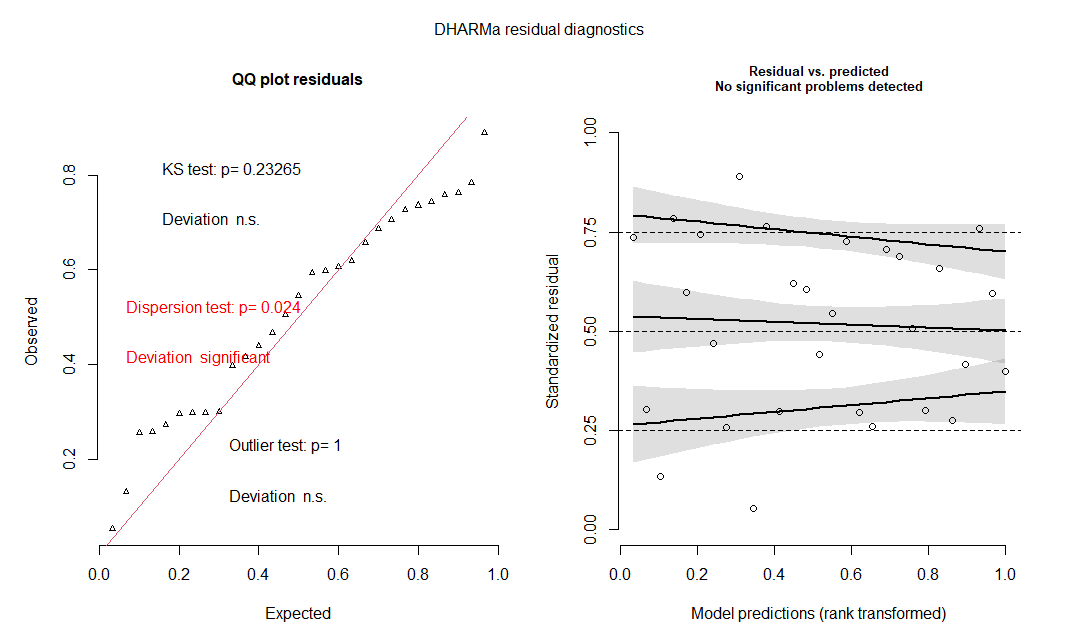
**

**Supplementary Figure S2** Predicted raccoon dog giving up densities relative to the interaction between wolf body odour and study period. Twenty was used as the upper limit for predicted GUDs and confidence intervals. The significance of the interaction was an artefact caused by the small sample size collected at plots without wolf scent in study period 1. This interactive effect was ultimately excluded from the final model explaining raccoon dog GUDs to reduce its complexity.


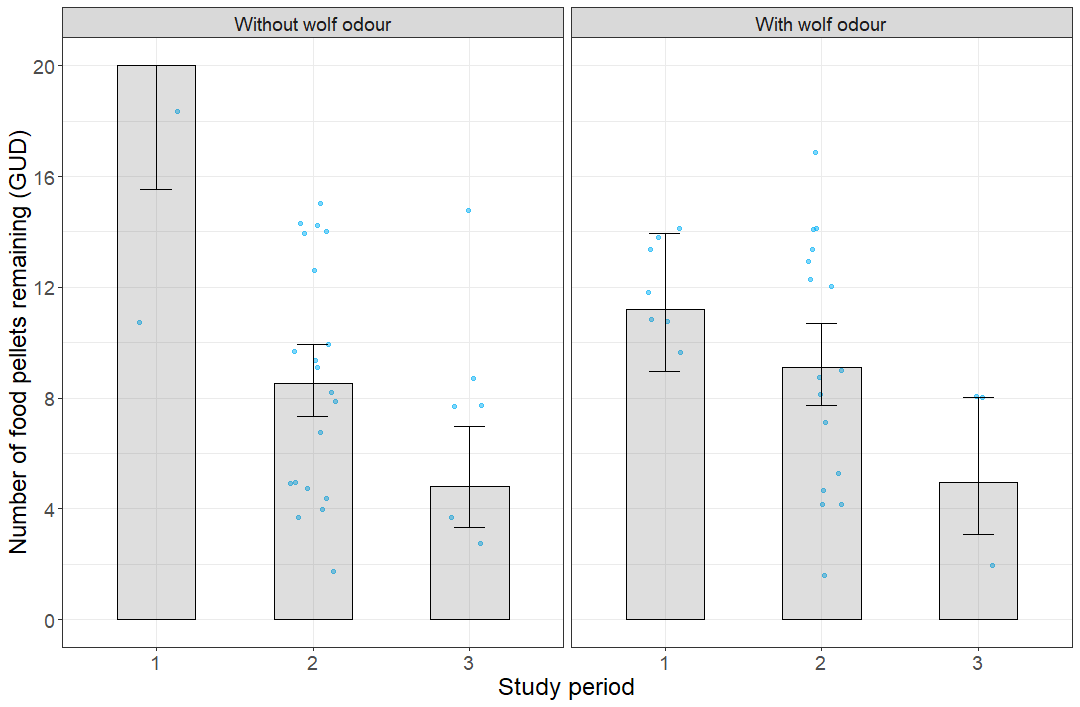


**Supplementary Figure S3** Predicted fox giving up densities relative to the interaction between wolf body odour and study period. Twenty was used as the upper limit for the confidence intervals. This interactive effect was ultimately excluded from the final model explaining raccoon dog GUDs to reduce its complexity.


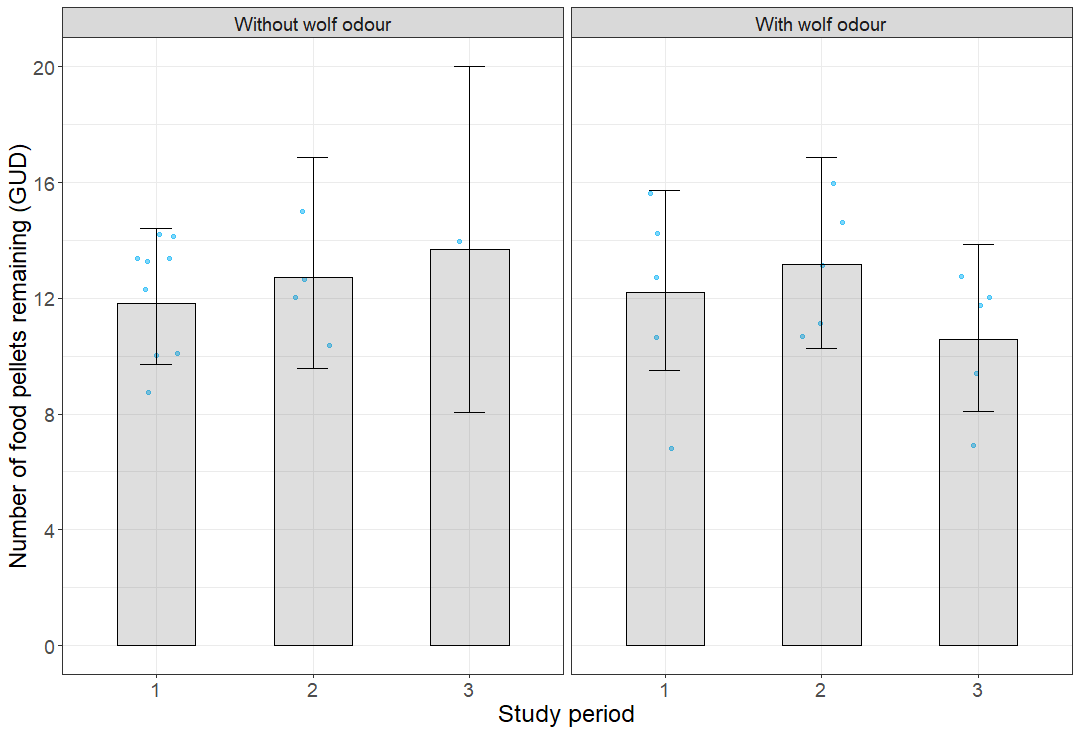


**Supplementary Figure S4** Raccoon dog GUD model diagnostic plots

**
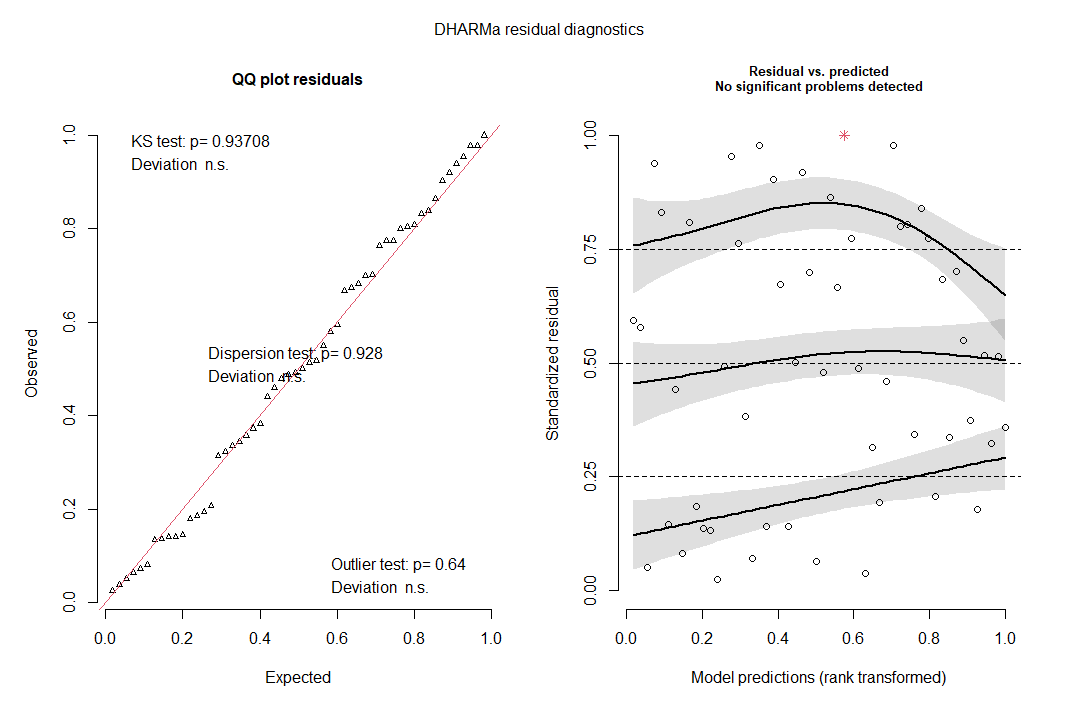
**

**Supplementary Figure S5** Raccoon dog study plot use diagnostic plots

**
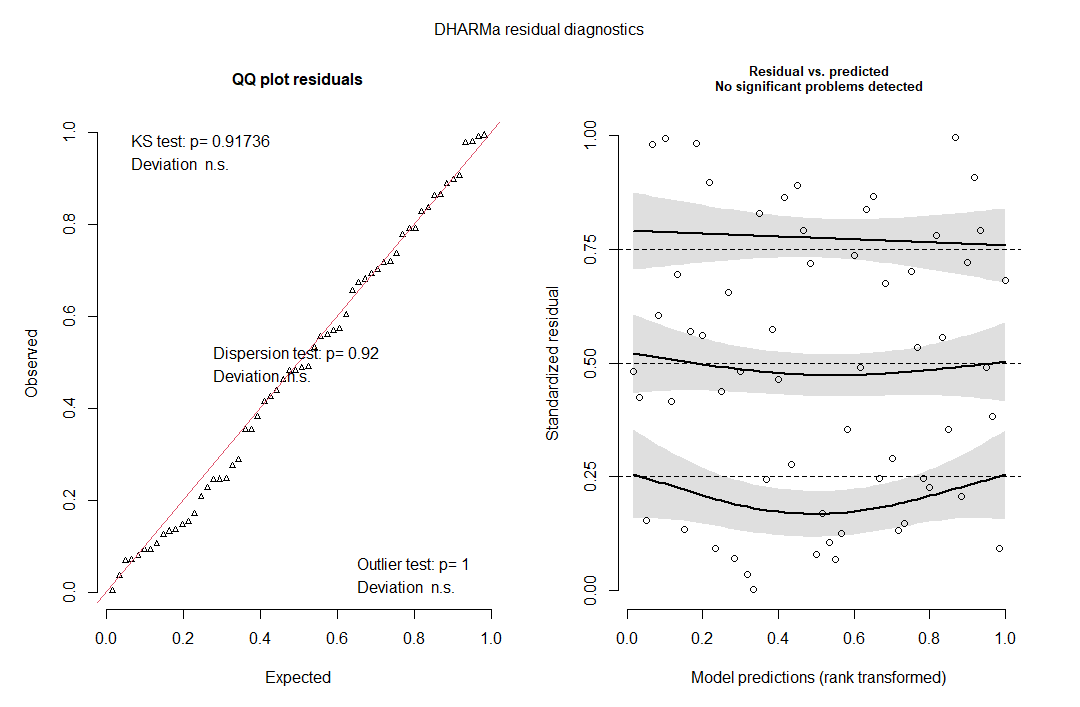
**

**Supplementary Figure S6** Fox study plot use model diagnostic plots


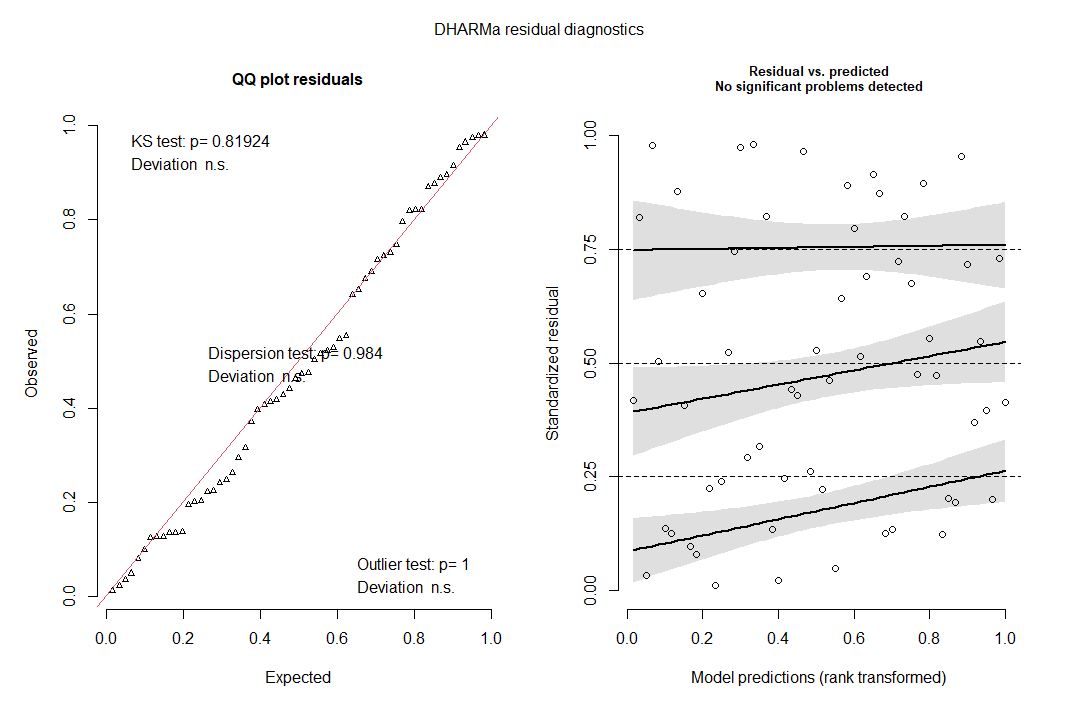


**Supplementary Figure S7** Predicted raccoon dog GUDs relative to study period. Error bars represent the 95% confidence intervals around the predicted values. Points represent the raw GUDs. Wolf encounter rates were based on the patterns of space use of wolves in 500 x 500 m grid cells around the study plots (based on Bubnicki et al., 2019). Study periods refer to different times in the year, 1 = Jun-Jul, 2 = Aug-Sept, 3 = October.

**
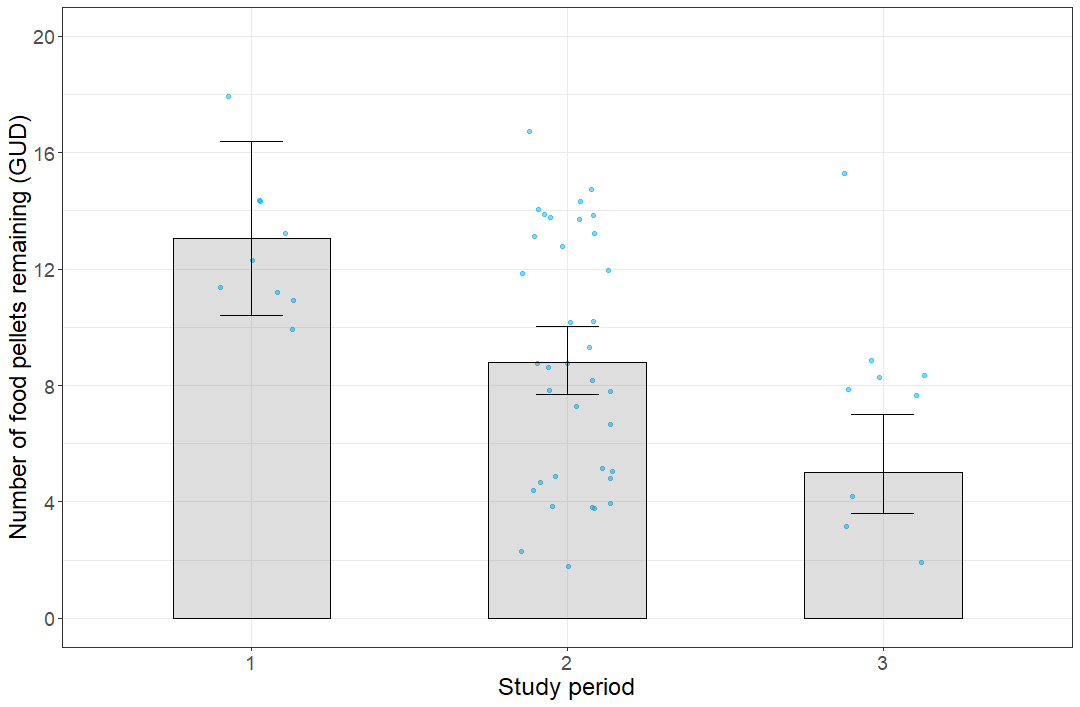
**

**Supplementary Figure S8** Predicted effect of the day of the session on raccoon dog giving up densities. Ribbons represent the 95% confidence intervals around the predicted values. Points represent the raw GUDs.

**
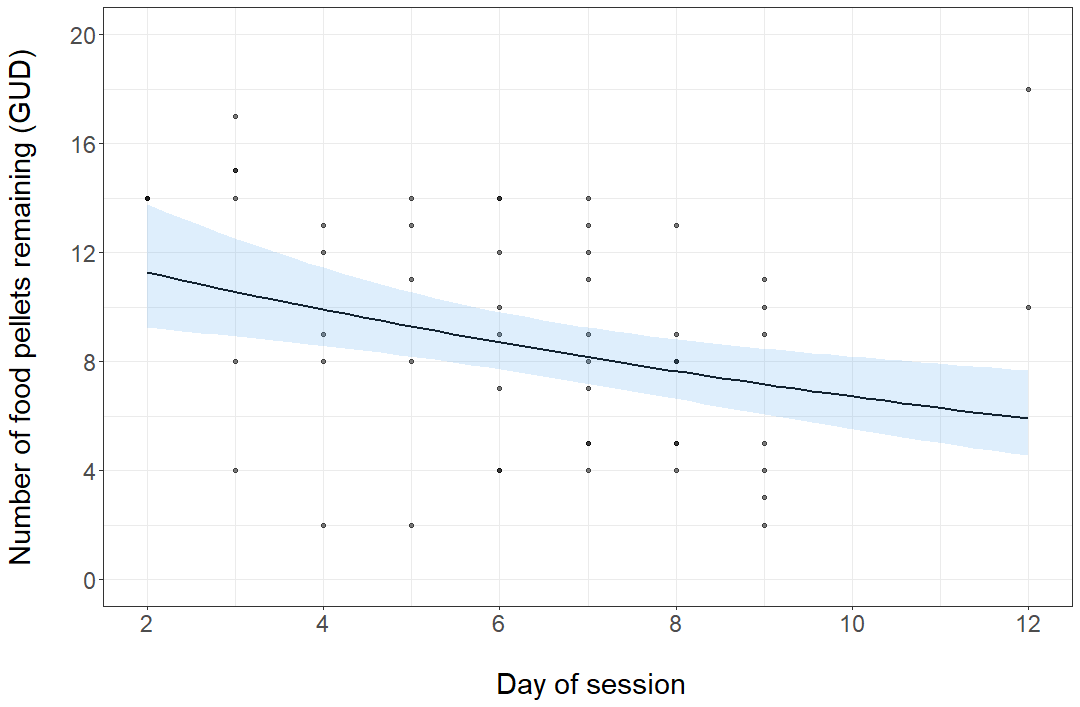
**

**Supplementary Figure S9** Predicted probability of study plot use by raccoon dogs in relation to study period. Error bars represent the 95% confidence intervals around the predicted values.


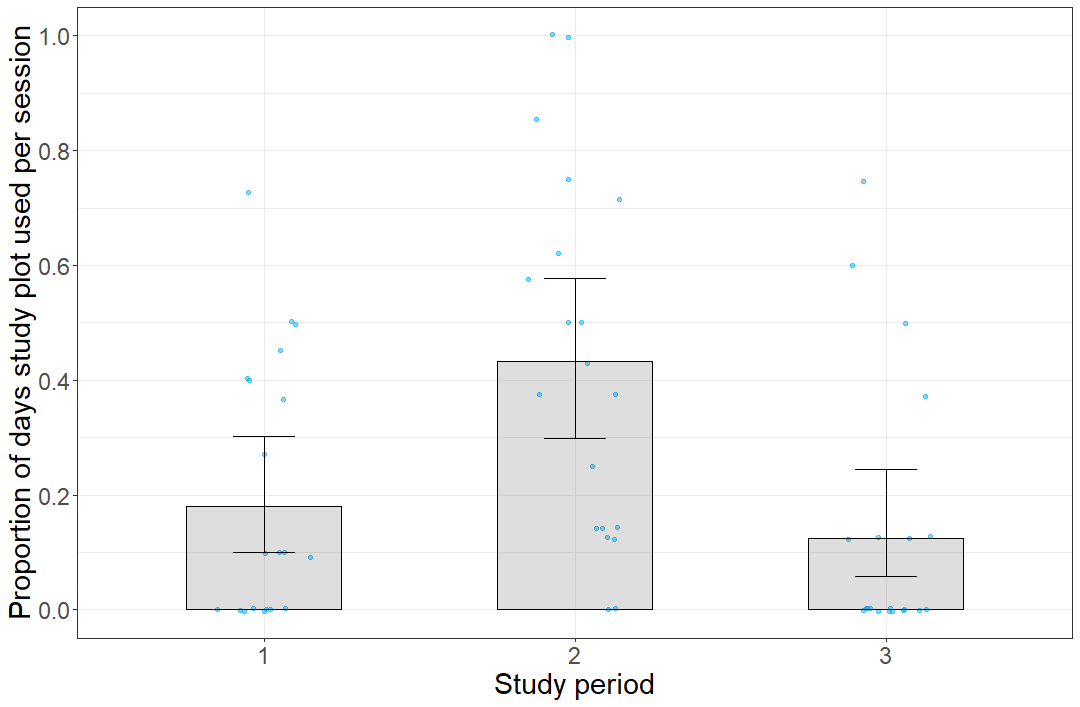


**Supplementary Figure S10** Predicted probability of study plot use by foxes in relation to study period. Error bars represent the 95% confidence intervals around the predicted values.

**
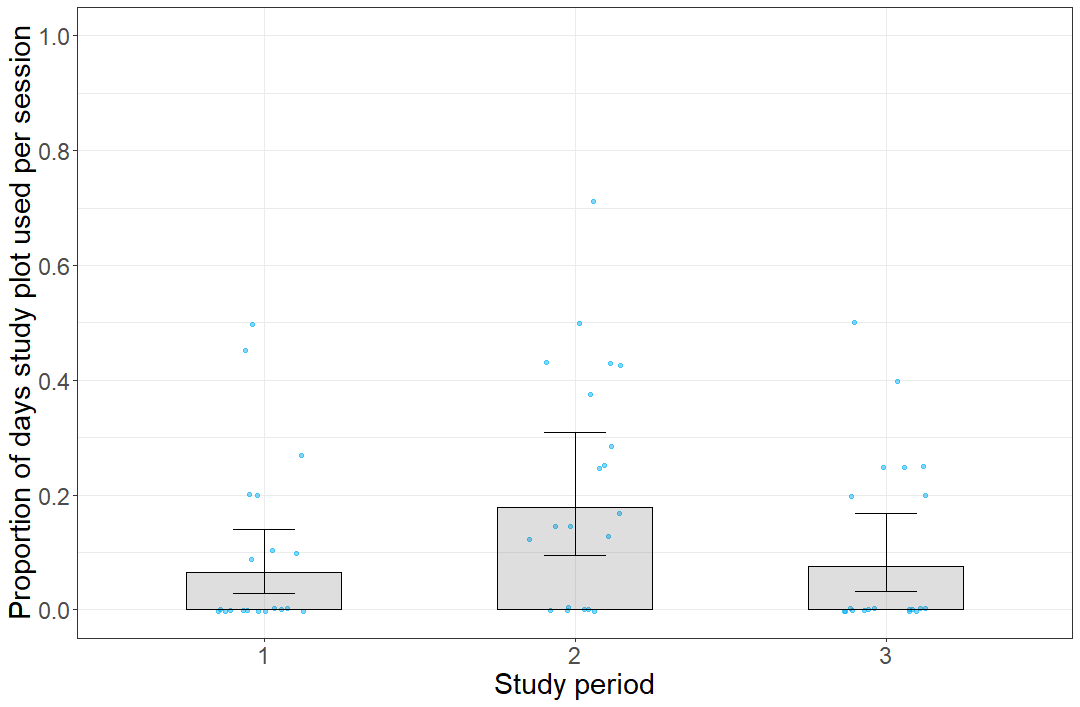
**
